# Differential performance of large language models in advanced cardiac life support assessment: A comprehensive multi-dimensional analysis of accuracy, consistency, and visual recognition capabilities

**DOI:** 10.1371/journal.pone.0347611

**Published:** 2026-04-29

**Authors:** Murat Genc, Bensu Bulut, Medine Akkan Öz, Ayşenur Gür, Mehmet Yortanlı, Betül Çiğdem Yortanlı, Oguz Sariyildiz, Ramiz Yazici, Hüseyin Mutlu, Zekeriya Uykan

**Affiliations:** 1 Department of Emergency Medicine, Ankara Training and Research Hospital, Ankara, Turkey; 2 Department of Emergency Medicine, Ankara Gulhane Training and Research Hospital, Health Science University, Ankara, Turkey; 3 Department of Emergency Medicine, Etimesgut Şehit Sait Ertürk State Hospital, Ankara, Turkey; 4 Department of Emergency Medicine, Konya Numune Hospital, Konya, Turkey; 5 Department of Internal Medicine, Konya City Hospital, University of Health Sciences, Konya, Turkey; 6 Department of Anesthesıology And Reanımatıon Department, 75th Year Oral And Dental Health Hospital, Ankara, Turkey; 7 Department of Emergency Medicine, Istanbul Kanuni Sultan Suleyman Training and Research Hospital, Health Science University, Istanbul, Turkey; 8 Department of Emergency Medicine, Aksaray Training and Research Hospital, Aksaray University, Aksaray, Turkey; 9 College of Engineering and Technology, American University of the Middle East, Egaila, Kuwait; PLOS, UNITED KINGDOM OF GREAT BRITAIN AND NORTHERN IRELAND

## Abstract

**Background:**

Large Language Models (LLMs) have been increasingly adopted in healthcare settings, yet comparative evaluations of their performance in standardized medical assessments remain limited. This study aims to evaluate the accuracy and consistency of four LLMs in answering Advanced Cardiac Life Support (ACLS) questions.

**Methods:**

In this observational study, 50 ACLS questions were categorized as knowledge-based (n = 29), visual content (n = 12), or case-based (n = 9). Each question was posed to ChatGPT-4o, Gemini 2.0, Claude 3.5, and DeepSeek R1 on three separate occasions to assess consistency. Performance was evaluated using three accuracy metrics: overall accuracy (all three responses correct), strict accuracy (at least two responses correct), and ideal accuracy (at least one response correct).

**Results:**

ChatGPT-4o demonstrated superior performance with 100% accuracy across all categories and perfect consistency (Fleiss’ Kappa = 1.0). Claude 3.5 achieved 92.0% overall accuracy with excellent consistency (Fleiss’ Kappa = 0.89). Gemini 2.0 showed 86.0% overall accuracy with moderate consistency (Fleiss’ Kappa = 0.58). DeepSeek R1 performed lowest at 70.0% overall accuracy with moderate consistency (Fleiss’ Kappa = 0.58) and failed completely on visual content questions (0%). All models achieved 100% accuracy on knowledge-based questions. Performance differences were statistically significant across models (p < 0.001).

**Conclusion:**

LLMs demonstrate variable capabilities in ACLS knowledge assessment, with ChatGPT-4o showing exceptional performance. While these models show promise as supplementary tools in resuscitation education and clinical decision support, significant variations in visual recognition capabilities and response consistency highlight the importance of critical evaluation before clinical implementation.

## 1. Introductıon

Cardiovascular diseases remain one of the leading causes of morbidity and mortality worldwide [1]. According to data from the American Heart Association (AHA), one person in the United States suffers a myocardial infarction every 40 seconds, and one death from cardiac arrest occurs approximately every four minutes [2]. These high incidence and mortality rates underscore the critical importance of timely and accurate management of cardiovascular emergencies [2,3]. In this context, Advanced Cardiac Life Support (ACLS) protocols represent the standard of care in the management of cardiac arrest and other acute cardiovascular events [3]. ACLS guidelines have been demonstrated to improve outcomes following cardiac arrest, whereas deviations from these protocols have been associated with decreased survival and poorer neurological recovery [4–6]. Improving outcomes after cardiac arrest requires structured, guideline-based and system-level quality improvement approaches [7].

ACLS training is a standardized educational program routinely provided to healthcare professionals and students, encompassing critical skills such as cardiopulmonary resuscitation (CPR), defibrillation, airway management, rhythm recognition, and pharmacological interventions. This training is conducted in alignment with guidelines that are typically revised every five years [6]. The theoretical component of ACLS certification is assessed through multiple-choice examinations that evaluate clinicians’ ability to make rapid and accurate decisions during cardiac emergencies [8]. In recent years, artificial intelligence (AI) and Large Language Models (LLMs) have gained increasing traction within healthcare systems. Owing to their natural language processing capabilities, these models can interpret, generate, and analyze medical texts effectively [9,10]. Prominent examples such as ChatGPT, Gemini, Claude, and DeepSeek have demonstrated promising potential in medical knowledge processing and clinical decision support [10]. A study by Aqavil-Jahromi et al. assessed the performance and reliability of GPT-3.5, GPT-4, Bard, and Bing chatbots in Basic Life Support (BLS) scenarios. The authors found that GPT-4 exhibited the highest accuracy—85%—in adult scenarios, whereas all models showed considerable limitations in pediatric and infant cases [11]. Similarly, research by Fijačko et al. indicated that ChatGPT achieved high relevance, accuracy, and compliance with resuscitation guidelines in life support assessments [8]. While prior studies have evaluated individual large language models in resuscitation-related scenarios, most have focused primarily on single-model accuracy or descriptive performance metrics. There remains limited evidence regarding inter-model comparison using repeated-measures designs, intra-model response consistency, and domain-specific performance differentiation within standardized ACLS examinations. Furthermore, the comparative evaluation of multimodal and non-multimodal models in image-based ACLS content has not been systematically addressed. To address these gaps, the present study provides a structured multi-dimensional assessment of four contemporary LLMs using repeated testing across three independent trials per question. Performance was analyzed using predefined accuracy criteria (overall, strict, and ideal consistency) and stratified according to question type (knowledge-based, case-based, and visual interpretation). By integrating reliability assessment with domain-specific and multimodal evaluation within a standardized ACLS framework, this study offers a more comprehensive characterization of LLM performance in advanced resuscitation knowledge assessment.

## 2. Materıals and methods

This observational study evaluated the response accuracy and consistency of four LLMs—ChatGPT-4o, Gemini 2.0, Claude 3.5, and DeepSeek R1—in answering questions from the ACLS module. The models’ comparative performance was assessed based on three levels of accuracy: overall accuracy, strict accuracy, and ideal accuracy. ACLS training has long been a standardized component of education for all healthcare providers, including students, with its guidelines typically updated every five years [3,6,12]. The ACLS certification exam consists of 50 multiple-choice questions, each offering five answer options. These questions are designed in alignment with the highest standards, drawing heavily from the AHA ACLS guidelines [13].

The question set used in this study was developed in accordance with the 2020 American Heart Association (AHA) Advanced Cardiovascular Life Support (ACLS) Guidelines, which were the most current officially implemented standards at the time of study design and data collection (September 2025). Four LLMs were utilized in this study. The first was ChatGPT-4o, also known as ChatGPT 4 Omni, which is currently widely reported to demonstrate strong performance in medical tasks [14,15]. The second model, Gemini 2.0, was selected based on prior evaluations of large language models in healthcare applications [16], and particularly due to reports suggesting superior performance in generating reliable referenced medical content [17]. The third model, Claude 3.5, was included based on reports indicating that its accuracy and output quality in medical tasks closely resemble that of human physicians [18]. Lastly, DeepSeek R1 was chosen for its reported potential as a promising AI tool in assisting with disease recognition and clinical condition classification [19].

A total of 50 multiple-choice ACLS questions from the 2020 exam were used in the evaluation. These questions were independently reviewed by two authors (R.Y. and A.G.) and categorized into three groups: knowledge-based, image-based, and case-based questions. This categorization was informed by established medical education assessment frameworks that distinguish between factual recall, applied clinical reasoning, and diagnostic interpretation domains, conceptually aligned with Bloom’s taxonomy [20,21]. In addition to educational assessment frameworks, this categorization was intended to reflect distinct cognitive components relevant to ACLS-related decision processes. Knowledge-based items primarily evaluate declarative knowledge of algorithms and pharmacological recommendations. Case-based questions require integration of contextual patient information and applied clinical reasoning. Visual items involve pattern-recognition processes, such as ECG interpretation, which are central to time-sensitive resuscitation scenarios. In instances of classification disagreement, a third author (H.M.) reviewed the item and made the final decision. Of the 50 questions, 9 (18%) were classified as case-based, 29 (58%) as knowledge-based, and 12 (24%) as image-based.

Between 12/06/2025 and 18/06/2025, each question was presented to the four models—ChatGPT-4o, Gemini 2.0, Claude 3.5, and DeepSeek R1—on three separate days by the same researcher (M.A.O.), using the same computer system. Each question was submitted in a separate, independent session to prevent contextual carryover between prompts. No prior responses were provided to the models, and no feedback was given between trials. The publicly available standard versions of the models were used without additional fine-tuning or custom training. As contemporary large language models do not update their core training parameters through individual user interactions within short-term sessions, the likelihood of within-study adaptive learning bias is considered minimal. All questions were entered verbatim into each model interface. The models were instructed to select the single best answer among the provided options (A–E) and to respond with the corresponding letter only. No additional contextual guidance, reasoning prompts, or step-by-step instructions were provided.Each model generated three responses per question. This protocol was designed to mirror previous studies assessing LLM response consistency and reliability by posing each item multiple times [8,11]. The performance of each model was assessed using three predefined accuracy criteria:

**Overall Accuracy**: A question was considered correct only if all three responses were accurate.

**Strict Accuracy**: At least two correct responses out of three were required for the answer to be considered correct.

**Ideal Accuracy**: A minimum of one correct response out of three qualified as a correct answer.

These complementary accuracy definitions were selected to differentiate between full response consistency (overall accuracy), majority agreement across repeated trials (strict accuracy), and partial correctness reflecting at least one accurate response (ideal accuracy). This approach allows for a structured evaluation of both response stability and potential answer variability across repeated testing. These classifications were designed to operationalize response consistency across repeated measurements and should be distinguished from inter-rater reliability, which was separately evaluated using Fleiss’ kappa statistics.

The official AHA key for ACLS questions and all model-generated responses were recorded in a dedicated Microsoft Excel file (Version 16.73, Microsoft Corporation, Redmond, WA, 2023). This study exclusively evaluated AI-generated responses and did not involve any human participants, access to medical records, or use of identifiable personal data. ACLS algorithms and educational content are widely disseminated in textbooks, review articles, and online educational resources. No patient-level data, biological samples, or protected health information were accessed. Therefore, institutional review board or ethics committee approval was not required in accordance with institutional and national regulations. Similarly, informed consent was not applicable.

### Statistical analysis

All statistical analyses were performed using SPSS version 27.0 (IBM Corp., Armonk, NY, USA). Categorical variables were expressed as frequencies and percentages (%). A two-sided alpha level of 0.05 was considered statistically significant. No missing data were present in the dataset. Differences in overall performance across language models were evaluated using Cochran’s Q test. Pairwise model comparisons were conducted using McNemar’s exact test with Bonferroni correction (adjusted significance threshold p < 0.0083). These comparisons were based on paired outcomes, as all models were evaluated using the same set of questions; therefore, McNemar’s test was preferred over the chi-square test. Response consistency across the three separate sessions was assessed using Fleiss’ Kappa statistic. Figures were generated using Microsoft Excel.

## 3. Results

In terms of overall accuracy, ChatGPT-4o demonstrated the highest performance with a perfect success rate of 100%. The other models showed comparatively lower accuracy rates: Claude 3.5 at 92.0%, Gemini 2.0 at 86.0%, and DeepSeek R1 at 70.0%. When evaluated under the strict accuracy criterion, ChatGPT-4o again achieved 100%, followed by Claude 3.5 with 94.0%, Gemini 2.0 with 92.0%, and DeepSeek R1 with 74.0%. Regarding ideal accuracy, ChatGPT-4o correctly answered all questions, maintaining its 100% success rate. Gemini 2.0 reached 96.0%, Claude 3.5 achieved 94.0%, and DeepSeek R1 maintained 74.0%.

Statistically significant differences were observed among the models across all three accuracy measures (p < 0.001) (Table 1).

**Table 1:** Comparison of overall and category-specific accuracy rates among large language models.

	ChatGPT 4o n (%)	Gemini 2.0 n (%)	Claude 3.5 n (%)	DeepSeek R1 n (%)	Cochran’s Q (df = 3)	p value*
**Overall accuracy**						
Total (n = 50)	50 (100.0)	43 (86.0)	46 (92.0)	35 (70.0)	27.923	<.001
Visual (n = 12)	12 (100.0)	8 (66.7)	10 (83.3)	0 (0.0)		
Knowledge (n = 29)	29 (100.0)	29 (100.0)	29 (100.0)	29 (100.0)		
Case (n = 9)	9 (100.0)	6 (66.7)	7 (77.8)	6 (66.7)		
**Strict accuracy**						
Total (n = 50)	50 (100.0)	46 (92.0)	47 (94.0)	37 (74.0)	22.560	<.001
Visual (n = 12)	12 (100.0)	10 (83.3)	11 (91.7)	0 (0.0)		
Knowledge (n = 29)	29 (100.0)	29 (100.0)	29 (100.0)	29 (100.0)		
Case (n = 9)	9 (100.0)	7 (77.8)	7 (77.8)	8 (88.9)		
**Ideal accuracy**						
Total (n = 50)	50 (100.0)	48 (96.0)	47 (94.0)	37 (74.0)	27.545	<.001
Visual (n = 12)	12 (100.0)	10 (83.3)	11 (91.7)	0 (0.0)		
Knowledge (n = 29)	29 (100.0)	29 (100.0)	29 (100.0)	29 (100.0)		
Case (n = 9)	9 (100.0)	9 (100.0)	7 (77.8)	8 (88.9)		

* Overall differences among the four language models were analyzed using Cochran’s Q test. Pairwise comparisons between individual models were performed using McNemar’s exact test with Bonferroni correction (adjusted significance threshold p < 0.0083).

Overal Accuracy: ChatGPT vs DeepSeek R1 (p < 0.001), Gemini 2.0 vs DeepSeek R1 (p = 0.008), Claude 3.5 vs DeepSeek (p = 0.003).

Strict Accuracy: ChatGPT vs DeepSeek R1 (p < 0.001), Claude 3.5 vs DeepSeek (p = 0.006).

Ideal Accuracy: ChatGPT vs DeepSeek R1 (p < 0.001), Gemini 2.0 vs DeepSeek R1 (p < 0.001), Claude 3.5 vs DeepSeek (p = 0.006).

**Fig 1:**
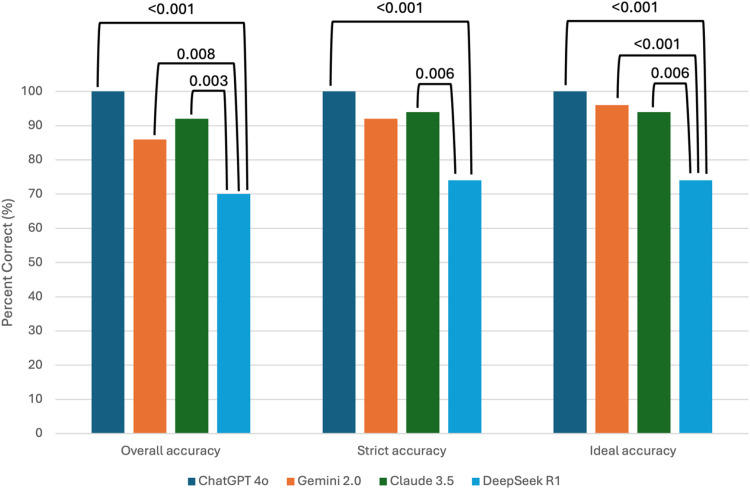
Comparative accuracy of large language models in ACLS question performance. Bar graph illustrating the percentage of correct responses provided by four large language models—ChatGPT-4o, Gemini 2.0, Claude 3.5, and DeepSeek R1—across three predefined accuracy criteria: overall accuracy (all three responses correct), strict accuracy (at least two responses correct), and ideal accuracy (at least one correct response). ChatGPT-4o achieved 100% in all accuracy metrics. Claude 3.5 and Gemini 2.0 also demonstrated high performance, while DeepSeek R1 exhibited significantly lower accuracy, particularly under the “overall” criterion. Statistically significant pairwise differences between models were calculated using McNemar’s test and are annotated above the bars (*p* values). All group comparisons were found to be statistically significant (*p* < 0.001).

When models were compared in pairwise analyses, statistically significant differences were particularly observed between DeepSeek R1 and the other models. The difference in overall accuracy between ChatGPT-4o and DeepSeek R1 was found to be statistically significant (p < 0.001). Similarly, significant differences were also identified between these two models in terms of strict accuracy and ideal accuracy (Table 1, Fig 1).

**Fig 2:**
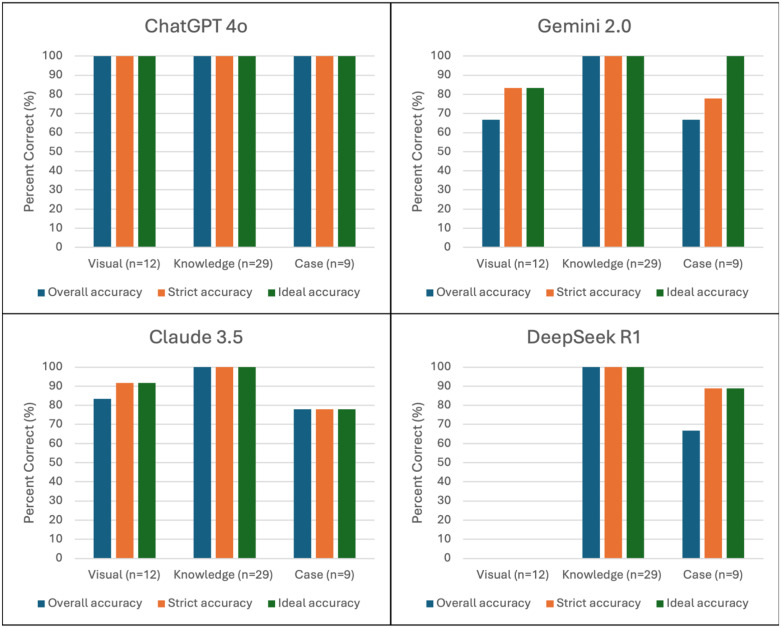
Performance percentages of large language models by question type. Bar charts displaying the accuracy rates of four large language models—ChatGPT-4o, Gemini 2.0, Claude 3.5, and DeepSeek R1—across three question categories: visual (n = 12), knowledge-based (n = 29), and case-based (n = 9). Each chart presents three predefined accuracy metrics: overall accuracy (all responses correct), strict accuracy (at least two correct responses), and ideal accuracy (at least one correct response). ChatGPT-4o achieved perfect performance (100%) across all question types and metrics. Claude 3.5 showed excellent accuracy in knowledge and visual items but relatively lower performance in case-based questions. Gemini 2.0 performed comparably in knowledge-based questions, but underperformed in visual and case-based categories. DeepSeek R1 achieved 100% accuracy in knowledge-based questions but failed to answer any visual question correctly and showed moderate performance in case-based items. These findings highlight model-specific variability in reasoning, domain knowledge, and visual recognition capabilities.

When performance was analyzed across specific question categories, ChatGPT-4o achieved a 100% accuracy rate on image-based questions, ranking first among all models. In contrast, DeepSeek R1 failed to correctly answer any of the image-based items. For knowledge-based questions, all models provided correct responses across the board. In the case-based category, ChatGPT-4o again ranked highest with 100% accuracy, while Claude 3.5 recorded the lowest performance with an accuracy rate of 77.8% (Table 1, Fig 2).

**Fig 3:**
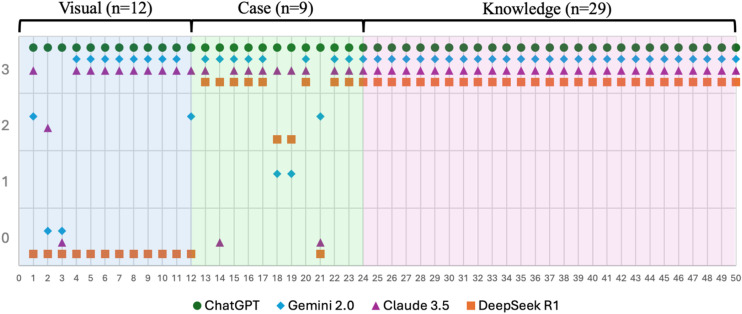
Response accuracy distribution of large language models across question types. Scatter plot depicting the distribution of response accuracy scores (0 to 3) for each of the 50 ACLS questions, categorized by question type: visual (n = 12), case-based (n = 9), and knowledge-based (n = 29). Accuracy scores were defined as follows: 3 = Strict accuracy (all three responses correct), 2 = Overall accuracy (at least two correct responses), 1 = Ideal accuracy (at least one correct response), 0 = No accuracy (all responses incorrect). Each symbol represents the performance of a different large language model—ChatGPT-4o (green circles), Gemini 2.0 (blue diamonds), Claude 3.5 (purple triangles), and DeepSeek R1 (orange squares)—on a specific question. ChatGPT-4o maintained perfect accuracy (score = 3) across all items, while DeepSeek R1 consistently failed (score = 0) in visual questions. Gemini 2.0 and Claude 3.5 exhibited variable performance, particularly in case-based and visual domains. The figure highlights performance heterogeneity across both question types and model architectures.

When the consistency of responses across three separate iterations of the same questions was evaluated, ChatGPT-4o demonstrated perfect agreement, providing correct answers to all questions in each trial. Claude 3.5 showed excellent consistency, with a Fleiss’ Kappa value of 0.89 (95% CI: 0.73–1.05, p < 0.001). In contrast, both Gemini 2.0 (Fleiss’ Kappa = 0.58, 95% CI: 0.42–0.74, p < 0.001) and DeepSeek R1 (Fleiss’ Kappa = 0.58, 95% CI: 0.40–0.77, p < 0.001) demonstrated moderate levels of agreement (Fig 3).

This figure illustrates the response accuracy distribution for each question across the four large language models (ChatGPT-4o, Gemini 2.0, Claude 3.5, and DeepSeek R1). For each question, models generated three responses, which were categorized as follows: Strict accuracy (3): All three responses were correct. General accuracy (2): At least two responses were correct. Ideal accuracy (1): At least one response was correct. Incorrect (0): The model consistently provided incorrect responses.

For each question, three responses were generated by each model. Accuracy categories were defined as follows:3- Strict Accuracy (all three responses were correct); 2 -Overall Accuracy (at least two responses were correct); 1 -Ideal Accuracy (at least one response was correct); 0 – No Accuracy (all three responses were incorrect). These categories are hierarchical and cumulative in nature, meaning that each level includes the criteria of the subsequent lower levels, with “ideal accuracy” representing the minimum acceptable threshold of correctness.

## 4. Dıscussıon

This study evaluated the accuracy and consistency of four LLMs—ChatGPT-4o, Gemini 2.0, Claude 3.5, and DeepSeek R1—in answering ACLS questions, highlighting the emerging role of artificial intelligence technologies in medical knowledge acquisition and clinical decision-making [19,22]. Our findings demonstrated that ChatGPT-4o achieved a 100% success rate across all accuracy metrics, while Claude 3.5, Gemini 2.0, and DeepSeek R1 achieved overall accuracy rates of 92.0%, 86.0%, and 70.0%, respectively. The ability of ChatGPT-4o to consistently provide correct answers in adult, pediatric, and infant scenarios underscores its potential utility in medical education and competency-based assessment. This result partially aligns with the study by Aqavil-Jahromi et al., which reported that GPT-4 achieved 85% accuracy in adult Basic Life Support (BLS) scenarios, but performed less reliably in pediatric and infant cases [11]. Similarly, Bushuven et al. found that ChatGPT and GPT-4 correctly identified pediatric emergency conditions and BLS scenarios in 94% of cases, but correctly recommended emergency services in only 54% of those cases [23]. In contrast, our study found that ChatGPT-4o demonstrated perfect accuracy (100%) in both adult and pediatric/infant scenarios, suggesting that this enhanced version represents a significant improvement in the model’s capacity for medical information processing. Although statistical significance was observed across models, the practical importance of these differences lies in their potential impact on reliability in educational and clinical contexts. In ACLS training environments, consistency of correct responses may influence learner trust and instructional utility. Conversely, variability across repeated responses may limit safe integration into high-stakes resuscitation workflows.

Beyond resuscitation-focused evaluations, recent studies have begun to assess the performance of large language models in broader medical and dental examination contexts. Tassoker et al. demonstrated that ChatGPT-4o achieved a 78% diagnostic accuracy in complex oral and maxillofacial pathology cases, indicating potential utility for clinical reasoning tasks, although performance varied according to case complexity [22]. Similarly, investigations conducted in dental specialty examination and anatomy-based assessment settings have reported heterogeneous performance profiles among contemporary LLMs, with accuracy varying across models, disciplines, and question types [24,25]. These findings underscore the influence of assessment structure and domain specificity on reported performance outcomes. Within this evolving literature, our study extends prior work by employing a multimodal, category-stratified evaluation within a standardized ACLS framework, combined with repeated measures for consistency analysis, thereby offering an integrated assessment of both accuracy and intra-model stability in a high-stakes emergency context.

In a study conducted by Altundağ et al., the performance of ChatGPT-3.5 in applying the AHA 2020 ACLS protocols was evaluated. The model achieved an accuracy of 86.3% on English questions and 81.3% on Turkish questions [26]. In comparison, our study demonstrated that ChatGPT-4o, a more recent model, achieved 100% accuracy, highlighting the substantial progress made by LLMs over time. While Altundağ et al. noted that ChatGPT-3.5 performed poorest in the “Class of Recommendation” section, our results showed that all models in the current study reached 100% accuracy in knowledge-based questions [26]. This suggests that LLMs are proficient in encoding static, terminology-driven content, yet performance differences become more apparent in tasks requiring clinical reasoning and contextual interpretation. In line with the structured design of our study, the differentiation of question types enables interpretation of model performance across distinct components of ACLS-related decision-making, including algorithmic recall, contextual reasoning, and pattern recognition. This stratified analysis provides a more comprehensive understanding of model capabilities beyond aggregate accuracy metrics. Notably, new-generation models such as ChatGPT-4o and Claude 3.5 outperformed less advanced models like DeepSeek R1 in tasks involving image recognition and complex contextual inference, further supporting the role of model sophistication in medical AI applications.

Another critical aspect contributing to the performance differences among AI models is their ability to process and interpret visual data. In a study by Zhu et al., GPT’s performance on Basic Life Support (BLS) exams was assessed, with an overall accuracy rate of 84%, which increased to 96% when open-ended questions were used [27]. However, notable variation has been observed in model performance when faced with visual scenario-based questions. Similarly, Chlorogiannis et al. highlighted the potential utility of language models like ChatGPT in the diagnosis and management of cardiovascular and cerebrovascular diseases, while emphasizing their limitations in processing visual information [10]. Beyond visual processing, performance variability has also been reported in complex, context-dependent emergency simulations. For example, Bushuven et al. reported that ChatGPT provided correct medical recommendations in only 40% of pediatric trauma cases based on simulated emergency prompts rather than direct visual input [23]. This finding suggests that even in text-based emergency scenarios, contextual reasoning may pose substantial challenges for language models [23]. In our study, ChatGPT-4o demonstrated 100% accuracy in answering image-based questions, followed by Claude 3.5 (83.3%) and Gemini 2.0 (66.7%), whereas DeepSeek R1 failed to answer any image-based questions correctly (0%). This disparity underscores the significant variability in visual processing capabilities among models. The observed variability across models further suggests that performance differences are more likely attributable to differences in model architecture and capability rather than simple memorization of fixed examination content. Notably, DeepSeek R1’s poor performance in image-based questions appears to be related to architectural constraints rather than deficiencies in medical reasoning. At the time of evaluation, the tested version of DeepSeek R1 did not support native multimodal input processing. Unlike multimodal architectures that integrate visual encoders with language models, the evaluated version operated primarily as a text-based system and was not designed to directly analyze visual content. Therefore, its low accuracy in image-based items likely reflects technical limitations in visual input handling rather than inadequate clinical knowledge.These findings have important implications for clinical scenarios requiring visual interpretation, such as electrocardiogram (ECG) analysis in cardiovascular emergencies, where the choice of AI model may directly influence diagnostic reliability.

In the study conducted by Aqavil-Jahromi et al., GPT-4 demonstrated a moderate level of response consistency (κ = 0.649), while Bard showed notably lower consistency (κ = 0.357) [11]. In contrast to their findings, our study observed substantially higher consistency levels across models. ChatGPT-4o achieved perfect agreement (Fleiss’ Kappa = 1.0) in all responses, supporting its potential as a reliable tool in clinical decision-making processes. Claude 3.5, which demonstrated excellent consistency (Fleiss’ Kappa = 0.89), also appears to be a viable alternative in healthcare settings. On the other hand, Gemini 2.0 and DeepSeek R1 displayed moderate consistency (both with Fleiss’ Kappa = 0.58), indicating that these models may require cautious use in clinical environments. Importantly, this finding illustrates that high accuracy does not necessarily imply high consistency. While Bignami et al. highlighted the transformative potential of natural language AI in emergency medicine, they also emphasized the associated risks and uncertainties that accompany its clinical integration [28]. Consistency is a critical factor, particularly in emergency medical settings, where the ability to deliver similar responses to the same clinical scenario is essential for reliability. From a clinical perspective, differentiating between strict and ideal accuracy provides insight into response stability versus occasional correctness. In high-stakes emergency scenarios such as ACLS, consistent generation of correct responses may be particularly relevant, as isolated correct outputs without reproducibility could pose potential risks in real-world application. In this context, ChatGPT-4o’s perfect consistency strengthens the case for its future application in clinical decision support systems (CDSS). However, given the life-threatening nature of cardiovascular emergencies, even occasional inconsistent outputs from LLMs may result in potential medical errors. Therefore, the use of such models in clinical practice should always be accompanied by expert medical supervision and judgment. It is important to emphasize that examination performance does not fully reflect real-world clinical competence. Human performance during resuscitation is influenced by cognitive load, team communication, situational awareness, and rapid adaptation to evolving clinical conditions. While LLMs operate without emotional stress, they also lack embodied clinical judgment, real-time environmental awareness, and responsibility for patient outcomes. Therefore, superior performance in standardized examinations should not be interpreted as superiority in real-life emergency care.

Another important consideration in medical education is the well-established discrepancy between factual knowledge and practical performance. Correctly answering multiple-choice questions does not necessarily translate into effective execution of cardiopulmonary resuscitation in real clinical settings, where psychomotor skills, teamwork, and situational adaptability are essential. Furthermore, the integration of AI tools into emergency medicine introduces an additional layer of competence: users must be adequately trained not only in clinical knowledge but also in interacting effectively with AI systems. The ability to formulate appropriate prompts, critically interpret model outputs, and recognize potential limitations of AI assistance constitutes an emerging form of clinical digital literacy. Therefore, the educational implications of AI integration extend beyond knowledge acquisition to include training in responsible and competent human–AI collaboration.

In their study, Preiksaitis et al. discussed the opportunities, challenges, and future directions of generative artificial intelligence in medical education, emphasizing its potential to transform the way medicine is taught and learned [29]. Similarly, Aqavil-Jahromi et al. noted that the performance of GPT models in Basic Life Support (BLS) assessments presents a promising avenue for supporting healthcare professionals in critical scenarios [11]. However, their study also highlighted the limited effectiveness of these models in pediatric and infant cases. In line with these concerns, Birkun et al. reported that both Bard and Bing performed poorly in scenarios involving non-breathing victims, failing to deliver guideline-concordant instructions [30]. One of the key takeaways from our study is the potential role of AI models in emergency medicine training and cardiopulmonary resuscitation (CPR) education. Given its high levels of accuracy and consistency, ChatGPT-4o may serve as a valuable educational tool for both medical students and healthcare professionals, supporting the acquisition and reinforcement of life-saving skills.

Beyond the training of healthcare professionals, LLMs may also hold value in educating and guiding the general public during medical emergencies. Blewer et al. emphasized that disparities in cardiopulmonary resuscitation (CPR) education remain a significant concern in the United States [31]. Supporting this, Bray et al. reported that CPR awareness and hands-only CPR knowledge among the general population are still insufficient [32]. Chlorogiannis et al. suggested that ChatGPT could play a role in patient education and symptom interpretation in cardiovascular disease contexts [10]. However, the study by Bushuven et al. revealed limitations in ChatGPT’s ability to understand and convey emergency instructions, with a correct first-aid instruction rate of only 45% [23]. These findings underscore the necessity of human oversight when using AI models for public health education and emergency communication. Nonetheless, the high performance demonstrated by ChatGPT-4o in our study suggests that next-generation models may offer greater reliability in this area, serving as valuable tools for increasing public awareness and delivering accurate health information during emergencies.

One of the main strengths of our study is that it provides a comprehensive evaluation of four distinct LLMs while being among the first investigations to include a wide range of question types—namely, knowledge-based, image-based, and case-based scenarios. Furthermore, presenting each question to the models on three separate occasions helped to increase the reliability of the consistency analysis. Nevertheless, our study has certain limitations. First, the evaluation relied exclusively on multiple-choice questions, which primarily assess factual recall and applied knowledge rather than real-world clinical performance. Therefore, the findings should be interpreted as a standardized model performance comparison rather than a direct indicator of real-world clinical competence. Future studies incorporating healthcare professionals and simulation-based or real-time clinical scenarios would provide a more comprehensive evaluation of LLM applicability in emergency medicine. Second, the evaluation was conducted on a total of only 50 ACLS questions, with 12 categorized as image-based, 29 as knowledge-based, and 9 as case-based. A broader item pool might have allowed for a more in-depth analysis of performance across varying levels of complexity. Although ACLS algorithms and guideline-based educational content are widely disseminated in textbooks and educational resources, the proprietary nature of large language model training corpora limits full transparency regarding potential exposure to similar materials. This possibility should be considered when interpreting model performance. Third, the scope was limited to four specific LLMs; other available AI systems were not included. Fourth, differences in multimodal processing capabilities among the evaluated models may have influenced comparative performance, particularly in image-based questions. At the time of evaluation, not all models supported native visual input processing. This architectural variability should be considered when interpreting cross-model comparisons, as performance disparities in image-based items may partly reflect technical constraints rather than differences in medical reasoning ability. Finally, since language models are continuously updated, our findings apply specifically to the model versions tested at the time of the study, and future iterations may produce different outcomes.

## 5. Conclusıons

In conclusion, this study demonstrates that ChatGPT-4o exhibited superior performance in ACLS-related knowledge, while Claude 3.5 and Gemini 2.0 achieved high levels of accuracy. In contrast, DeepSeek R1 showed limited capability, particularly in handling image-based questions. These findings support the potential role of LLMs in cardiovascular emergency care, while also highlighting the importance of acknowledging their current limitations. The integration of LLMs into clinical education, simulation training, and decision support systems may contribute meaningfully to advancements in resuscitation medicine; however, such applications must be supplemented by clinical expertise and human oversight. Future studies should focus on evaluating model performance in real-time clinical scenarios, assessing the interpretability and clarity of responses for patients, and exploring performance variability across different medical specialties.

## Supporting information

S1 File Data. (XLSX)
